# Effects of a Mixed Starter Culture on the Biogenic Amine Accumulation, Volatile Flavor, and Quality of Jinhua Dry‐Cured Ham

**DOI:** 10.1002/fsn3.70324

**Published:** 2025-06-12

**Authors:** Lin Chen, Lu Huang, QingYu Zhang, WuMei Yu

**Affiliations:** ^1^ Department of Food Research Zhejiang Business College Hangzhou China

**Keywords:** biogenic amine, dry‐cured, Jinhua ham, volatile flavor

## Abstract

Jinhua ham is a traditional dry‐cured product that favors an indigenous microflora, crucial for sensory characteristics. However, spontaneous fermentation may yield toxins such as biogenic amines. This study evaluates the impact of a mixed starter culture containing *Lactiplantibacillus plantarum, Lactilactobacillus sakei,* and *Staphylococcus xylosus* (*LLS*) on microbial diversity, biogenic amines accumulation, volatile flavor, quality, and sensory attributes of Jinhua dry‐cured ham. Microbial profiling revealed that within the *LLS* starter culture, the *Lactobacillaceae* and *Staphylococcaceae* species predominated, with their relative abundances ranging from 15.2% to 42.3% and 3.5% to 35.6% throughout the fermentation process, respectively. The *LLS*‐enriched ham demonstrated marked reductions in biogenic amines such as histamine, putrescine, tyramine, and cadaverine, accompanied by decreases in pH, water activity (*a*_*w*_), and total volatile basic nitrogen (*p* < 0.05) compared to the control. Using solid‐phase microextraction coupled with gas chromatography–mass spectrometry, 56 volatile flavor compounds were detected in the Jinhua ham. The ham treated with *LLS* showed an increment of 11 volatile species compared to the control, enhancing the presence of desirable volatiles including 2,6‐dimethylpyrazine, 2‐ethyl‐1‐hexanol, and 1‐octen‐3‐ol. These findings suggest that *LLS* starter cultures may serve as effective agents in Jinhua dry‐cured ham production to mitigate BA formation while enhancing sensory properties.

## Graphical abstract

A compound starter culture consisting of *L. plantarum* , *L. sakei* , and *S. xylosus* has the potential to reduce BAs and enhance sensory quality in Jinhua ham.

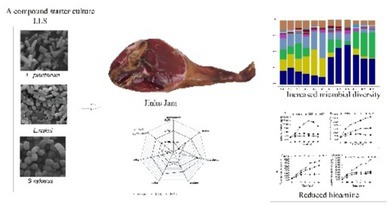

## 1 Introduction

Jinhua ham, a distinguished traditional dry‐cured delicacy originating from the Jinhua region in Zhejiang province, is compared with other dry‐cured hams, which are produced from the hind legs of the Liangtouwu breed, celebrated for its diverse microbiota and distinctive flavor (Huan et al. 2005; Du and Ahn 2001). This ham undergoes a lengthy natural fermentation period of 8–10 months (Miao et al. 2009), akin to European dry‐cured hams (Jiang et al. 2022). While fermentation by indigenous microflora enhances flavor and nutrient content, it also leads to the production of potentially harmful substances, such as biogenic amines (BAs) (Liu et al. 2018). BAs, nitrogen‐containing, low‐molecular‐weight, non‐volatile bioactive compounds, are prevalent in fermented foods, particularly meats (Nie et al. 2014). Although BAs play roles in regulating nervous activity and promoting cell growth and metabolism, excessive intake beyond the body's degradation capacity—particularly of histamine, putrescine, tyramine, and cadaverine—may damage the nervous system and cause abdominal cramps and breathing disorders. Current strategies for BA inhibition in dry‐cured ham involve modifying production processes, adjusting sterilization methods, and adding antibacterial agents; however, these can impair the quality and flavor of the ham (Mohammed 2016; Komprda et al. 2007). Therefore, the development of safe starter cultures that are both effective and have minimal adverse effects is a critical area of research in dry‐cured ham production.

Traditional Jinhua hams, typically fermented spontaneously with indigenous microflora, often suffer from contamination by undesirable microbes, leading to inconsistencies with product safety and quality standards. In most countries, lactic acid bacteria (LAB) are regarded as natural and safe starter cultures for fermented meat products (Wang et al. 2015; Woraprayote et al. 2016); however, the vast majority of studies focused on sausage, and the dry‐cured ham starter culture was rare, especially for Jinhua ham. It has been demonstrated that the strains isolated from indigenous microorganisms in fermented food could reduce BAs accumulation. Fifty strains of *Staphylococcus xylosus* ( *S. xylosus* ) with amine oxidase activity from Italian sausages, of which six strains were able to reduce histamine levels by up to 90% (Martuscelli et al. 2010). *Lacticaseibacillus casei*, as a competitive co‐fermenter, significantly controlled BAs during ripening for cheeses (Herrero‐Fresno et al. 2012). Moreover, a suitable starter culture of fermented meat could contain compound strains; in addition to biodegradable BAs strains, it is great to explore for extending stability and shelf life (Komprda et al. 2007). *Staphylococcus*, used as a starter culture for food manufacturing‐fermented and bio‐preserved foods primarily enhance flavor and maintain stability. *Lactiplantibacillus plantarum* NJAU‐01 has been shown to significantly enhance color and texture in ham (Ge et al. 2019). The inoculation of *Lactiplantibacillus plantarum* MSZ2 and *S. xylosus* YCC3 inhibited harmful bacteria in fermented sausages and improved storage period (Wang, Aziz, et al. 2022; Wang, Hou, et al. 2022). Additionally, the presence (Talon and Zagorec 2017) of *Lactiplantibacillus plantarum* R2 and *S. xylosus* A2 is believed to enhance the sensory quality of fermented meat products by facilitating protein hydrolysis and fat decomposition (Xiao et al. 2020).

The strains of starter culture play a critical role in the flavor formation of dry‐cured ham. Quality and flavor of ham depend on microorganisms and endogenous enzymes. The microbial flora during ham fermentation changed by the conditions of raw materials and environment directly affect flavor and nutritional value (Wang, Li, et al. 2021; Wang, Shen, et al. 2021). The starter culture research shows that *Lactiplantibacillus plantarum*, *Lactilactobacillus sakei*, and *Weissella hellenica* ( *W. hellenica* ) are the dominant strains in fermented meat associated with the production of esters, aldehydes, and ketones (Hu et al. 2020). It has been proved that the mixed starter culture of *Lactiplantibacillus plantarum* YR07, *Lactilactobacillus sakei* L.48, *S. xylosus* S.14, and *Mammaliicoccus sciuri* S.18 is efficient in improving characteristic flavor compounds such as 3‐hydroxy‐2‐butanone, hexanal, and 1‐octen‐3‐ol in fermented sausages (Zheng et al. 2024). Our previous study indicated that the *S. xylosus* M06 screened from indigenous microorganisms of Jinhua hamcould effectively reduce BAs; however, this strain separately fermented in Jiahua ham accompanied with short shelf‐life and undesirable flavor. *Lactiplantibacillus plantarum* ZJ316 in our lab has been identified for its production of Plantaricin, exhibiting significant antimicrobial effects against both Gram‐positive and Gram‐negative bacteria, which significantly enhances pork quality. *Lactilactobacillus sakei* Z18 cultured from fermented sausage could improve the flavor and meat texture (Chen et al. 2018; Suo et al. 2012). Based on prior research, the aim of this study was to investigate an innovative mixed starter culture, explore the strains synergistic effect, appraise its application on bacterial profile, BAs, volatile flavors, and quality of hams, and provide a potential commercial starter for Jinhua dry‐cured ham.

## 2 Materials and Methods

### 2.1 Starter Culture and Chemicals

*Lactiplantibacillus plantarum* ZJ316, derived from the fecal samples of healthy newborns, along with *S. xylosus* M06, isolated from Jinhua ham, and *Lactilactobacillus sakei* Z18 sourced from Chinese fermented meats—were obtained from the Key Laboratory for Food Microbial Technology of Zhejiang Province, China. The *LLS* starter culture, comprising these three strains in equal ratios (m/m) of 1:1:1, was cultured to a density of 10^9^ CFU/g and prepared as a freeze‐dried powder. The commercial S015 starter culture, consisting of *Debaryomyces hansenii* and *Staphylococcus xylosus* used for fermented meats, was acquired from the Research Center for Meat Processing and Quality Engineering Technology of Zhejiang Province, China. Histamine, putrescine, tyramine, cadaverine, and the internal and standard compounds for volatile identification were purchased from Sigma Aldrich (Sigma, USA).

### 2.2 Jinhua Ham Preparation and Sampling

Three batches of Jinhua hams were prepared: (1) a control group without starter culture (control); (2) hams inoculated with the *LLS* starter culture (*LLS*); (3) hams inoculated with the commercial S015 starter culture (S015). The hams were produced using modern techniques at the Jinhua Ham Corporation, Zhejiang, China. Fresh hind legs weighing 6.0–7.0 kg from Lanxi (Zhejiang province), obtained from 4 to 6 month‐old Liangtouwu crossbreeds weighing 80–90 kg, were used. The fresh hams were stacked and salted at 75%–85% relative humidity and 2°C–8°C for 28 days; sodium chloride was added in three stages to achieve a total of 5.5% by weight. After dry‐curing, the hams were washed in 15°C water for 24 h, then inoculated with the different starter cultures and transferred to a fermentation room for 60 days, maintaining fermentation conditions at 20°C–25°C and 70%–75% humidity. After fermentation, the samples were ripened for 7 days. Sampling of 100 g of ham for BA analysis, physicochemical properties, LAB, and bacteria counts was carried out on days 0, 10, 20, 30, 40, 50, and 60, in triplicate. Microbiological analysis was conducted on days 10 and 60.

### 2.3 Microbiological Analysis

The microbial composition of Jinhua ham was characterized using advanced high‐throughput sequencing techniques. Total DNA from the ham samples, both control and *LLS*‐treated, on days 10 and 60, was extracted using the E.Z.N.A. Mag‐Bind Soil DNA Kit (OMEGA, USA). Agarose gel electrophoresis was used to assess the DNA's amount and integrity. After the bacterial 16S rRNA gene's V4 region was quantified, the following primers were used to amplify it: 515F (5′‐GTGCCAGCMGCCGCGGTAA‐3′) and 805R (5′‐GGACTACHVGGGTWTCTAAT‐3′). Purification with Beckman DNA Clean Beads and measurement with a Qubit 2.0 fluorimeter (Invitrogen, Carlsbad, CA, USA) were the subsequent steps after the PCR products were validated on a 1% agarose gel (Invitrogen, Carlsbad, CA, USA) (Qiu et al. 2018). Following conventional methods, these purified products were sequenced on an Illumina HiSeq 2500 system (Illumina, San Diego, CA, USA). Operating taxonomic units (OTUs) were formed by clustering sequences that displayed a similarity level of 97% or above. Total OTUs were normalized by comparing them to the sample that had the fewest sequences. Principal Coordinate Analysis (PCoA) was used to evaluate differences in microbial communities, and QIIME (Version 1.7.0) alpha diversity indices were used to quantify diversity within the samples. R software (Version 2.15.3) was used to examine beta diversity and inter‐group variations (Bokulich and Spiller 2016). The LAB and total bacteria counts were assessed by standard plate count method.

### 2.4 Determination of BAs

BA quantification was adapted from Sun et al. (2016) and conducted using high‐performance liquid chromatography (HPLC). A solution of standard amines was produced in 0.4 M perchloric acid, with concentrations ranging from 0.0 to 20.0 mg/mL. Twenty milliliters of 0.4 M perchloric acid was used to homogenize a 10 g sample of Jinhua ham, which was then centrifuged at 8000× g for 10 min at 4°C. After three extraction cycles, the combined supernatants were adjusted to a total volume of 50 mL. The 1 mL of this extract, 100 μL of 1.5 M sodium hydroxide, 2 mL of 10 mg/mL dansyl chloride (Acetone solvent), and 250 μL of saturated sodium bicarbonate that made up the analytical sample was left to incubate at 50°C for 20 min in the absence of light. Following the incubation period, 100 μL of ammonium hydroxide (1.0 mol/L) was introduced, and 5 mL of acetonitrile was used to increase the volume. Subsequently, the mixture was filtered through a 0.22 μm membrane. With a temperature maintained at 40°C and a flow rate of 1.0 mL/min, chromatographic analysis was carried out using a ZORBAX Extend‐C18 column (250 × 4.6 mm, 5 μm) at 254 nm. The gradient elution program started with 60:40 (v/v) acetonitrile: water at a flow rate of 1.0 mL/min, followed by a linear increase to 85:15 (v/v) acetonitrile: water (1.0 mL/min) during the next 3 min, and then a linear increase to 100% acetonitrile (1.0 mL/min) for 10 min, and the ratio of acetonitrile: water was decreased to 60:40 (1.0 mL/min) for the last 10 min.

### 2.5 pH, a_w_, and Total Volatile Bases Nitrogen (TVB‐N) Value

pH levels were assessed using an electronic pH meter (Mettler Toledo FE28), while water activity (*a*_*w*_) was evaluated using an AquaLab 4TE meter (USA). TVB‐N concentrations were quantified by semi‐micro distillation. A 10 g ham sample was homogenized with sterile water for 20 min; the mixture was filtered to collect the extract, to which 10 g/L magnesium oxide was added, and then steam distilled. The volatile alkaline components were absorbed by a borate receiver and quantified by titration.

### 2.6 Color and Texture Properties Analysis

Color properties of the ham were analyzed using a colorimeter Spectro‐pen (HunterLab MiniScan 45/0 LAV, USA), which measures the CIE L* (lightness), a* (redness), and b*(yellowness). Texture Profile Analysis (TPA) was conducted using a texture analyzer (TMS‐Pro, FTC, USA). Ham samples were cut into 1 cm^3^ cubes and analyzed for hardness and springiness using a cylindrical probe with a diameter of 35 mm (p/50).

### 2.7 Determination of Volatile Flavor

Using gas chromatography–mass spectrometry (SPME‐GC–MS), we were able to analyze the fermented Jinhua hams for volatile taste components. An internal standard of 20 ng of deuterobenzene was added to a 20 mL sealed vial containing 3 g of ground ham. The 50/30 μm DVB/CAR/PDMS SPME fiber was used to extract volatiles. After 50 min in a 65°C water bath, the fiber was exposed to the sample vial for 40 min. After that, the fiber was subjected to a 5‐min desorbed period in the GC injector at 250°C. The TG‐624SilMS column, measuring 60 m × 250 μm × 1.4 μm, was used for chromatographic separation. Helium was used as the carrier gas, and the flow rate was set at 1.0 mL/min. The amount of volatile chemicals was determined by comparing their peak regions to the internal standard (Atallah 2021).

### 2.8 Sensory Evaluation

Ham samples were cut into 2 mm thick slices for sensory analysis. Sensory descriptors, including appearance, flavor, hardness, texture, color, odor, and overall appearance, were assessed using a modified version of the method with some modifications (Cavalheiro et al. 2019). Prior to analysis, the ham samples from normal and spoiled hams were thinly sliced and placed at room temperature no more than 10 min before being served. Twenty assessors were trained to enable trainees to understand the test procedure. The ham samples were coded numerically and presented to 20 assessors randomly. Acceptability was rated on a six‐point scale from 0 to 10 by the assessors. The value of 0 corresponded to the lowest and the value of 10 to the highest for the six sensory factors. The sensory tests are conducted in a sensory laboratory equipped with separate compartments.

### 2.9 Statistical Analyses

Statistical analysis was undertaken using GraphPad Prism version 10.1. Differences between groups were evaluated using one‐way ANOVA, with results presented as mean ± standard deviation (SD), and significance determined at *p* < 0.05.

## 3 Results and Discussion

### 3.1 Effect of the Starter Culture on Bacterial Diversity

**FIGURE 1:**
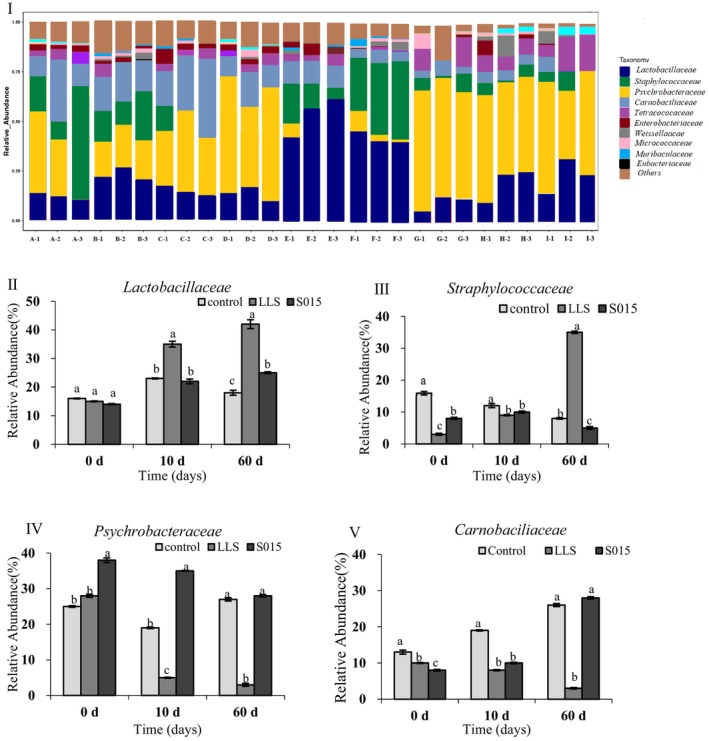
Relative abundances of main family in the different Jinhua hams. A: The control group, Day 0; B: The control group, Day 10; C: The control group, Day 60; D: The LLS group, Day 0; E: The LLS group, Day 10; F: The LLS group, Day 60; G: The S015 group, Day 0; H: The S015 group, Day 10; (I) The S015 group, Day 60. The microbial communities of different Jinhua hams. (II) *Lactobacillaceae*; (III) *Staphylococcaceae*; (IV) *Psychrobacteraceae*; (V) *Carnobaciliaceae*. Different letters indicate significant differences in different groups at the same stage (*p* < 0.05).

The bacterial relative abundance in four ham samples was analyzed using high‐throughput sequencing. The samples included Day 0, 10, and 60 in the control, LLS, and S015 group. Annotation at the family level revealed a total of 10,825 gene sequences with identifiable genera (Figure 1). In the control group, *Psychrobacteraceae* (31.8%) and *Carnobaciliaceae* (24.6%) were predominant, whereas *Lactobacillaceae* (43.5%) and *Staphylococcaceae* (26.8%) dominated in the *LLS* and S015 starter culture. Dry‐cured ham undergoing spontaneous fermentation is vulnerable to pathogenic bacteria, which can cause fat oxidation, odor development, decomposition, and BAs production. Differences in microbial diversity were observed between the treatments. *Psychrobacteraceae* and *Carnobaciliaceae*, known as spoilage bacteria, produce metabolites that alter the color and viscosity of the meat. *Lactobacillaceae*, on the other hand, can produce bacteriostatic substances during fermentation, helping to regulate the microbial balance. These results corroborate findings by Wang, Li, et al. (2021) and Wang, Shen, et al. (2021) that *Staphylococcus* and *Lactobacillaceae* predominate in the microbial communities of dry‐cured hams, with *Staphylococcus* showing a notably higher relative abundance in Jinhua ham compared to Rugao and Xuanwei hams. In this study, the S015 exhibited a high relative abundance of *Psychrobacteraceae* during fermentation; for the *Carnobaciliaceae*, the relative abundance reached the highest in the later stage. In the control group, the relative abundance of *Staphylococcus* decreased, while *Carnobaciliaceae* abundance increased with fermentation time. However, in the *LLS* starter culture group, the relative abundance of *Lactobacillus* and *Staphylococcus* ranged from 15.2% to 42.3% and 3.5% to 35.6%, respectively. These results suggest that LAB starter cultures could enhance safety and improve the sanitary quality of products in the industry. *Lactiplantibacillus plantarum* ZJ316 in the LLS starter culture has been identified with bacteriocin‐producing ability. *Lactilactobacillus* and *Staphylococcus* could inhibit the growth of pathogens and spoilage bacteria through acidification or the production of antibacterial agents (Chen et al. 2018).

**FIGURE 2:**
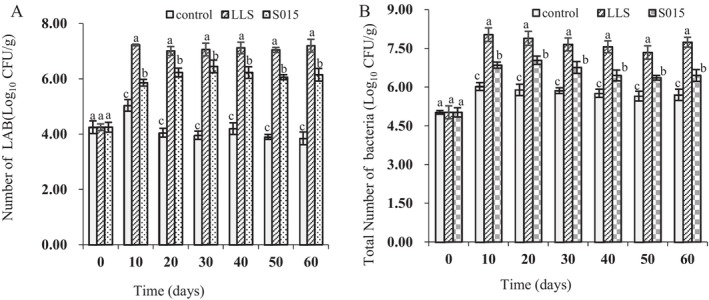
Bacteria counts in the different Jinhua hams. (A) LAB counts; (B) Total bacteria counts. Different letters indicate significant differences in different groups at the same stage (*p* < 0.05).

The LAB and total bacteria counts in different ham groups were shown in Figure 2. With the extension of fermentation time, the total bacteria colonies of the groups showed an upward trend. After 10 days, the counts began to decrease; the total number of bacteria in LLS and S015 was significantly higher than in the control group. In the fermentation process, the LAB in starter culture was the dominant bacteria group, exhibiting a significantly higher number than the control group. The LLS group showed the highest value on Day 10 and the S015 group on Day 30. Compared with the S015 group, the LAB counts of the LLS group were significantly increased in fermentation time (*p* < 0.05).

### 3.2 Effect of Starter Culture on BA Formation

**FIGURE 3:**
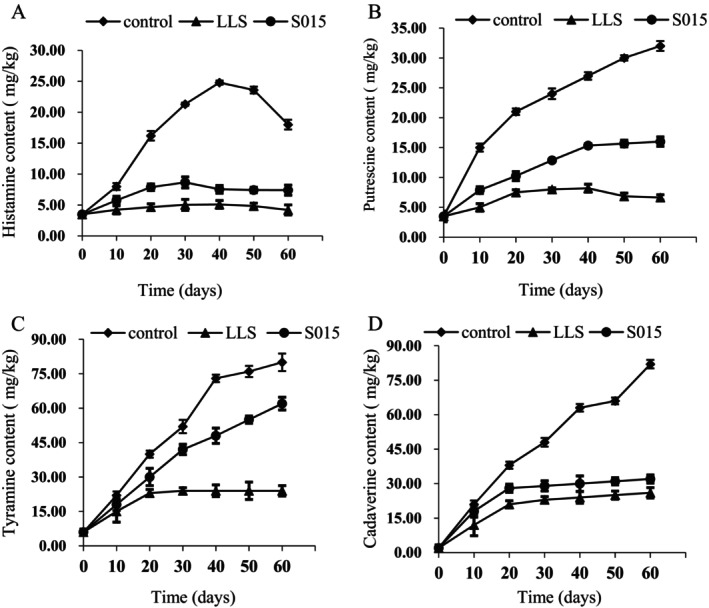
Effects of starter cultures inoculation on histamine (A), putrescine (B), tyramine (C), and (D) cadaverine accumulation in Jinhua ham.

**FIGURE 4:**
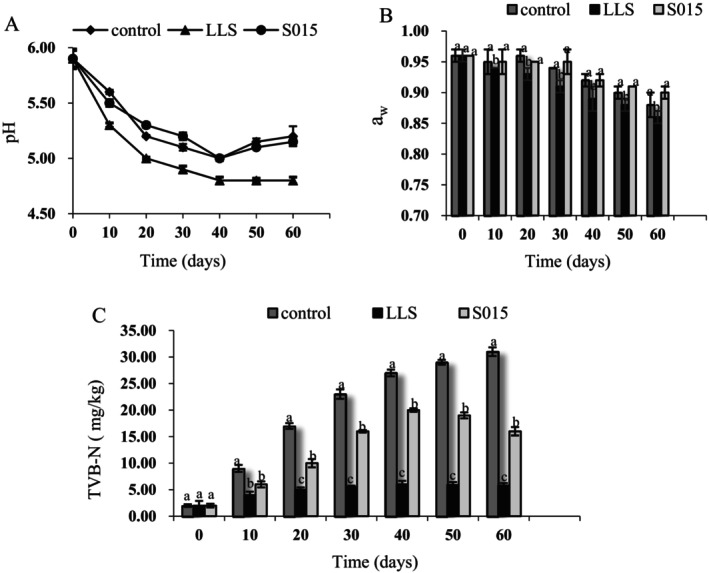
Effects of starter cultures inoculation on pH (A), water activity (B), TVB‐N content (C). Different letters indicate significant differences in different groups at the same stage (*p* < 0.05).

The impact of various starter cultures and spontaneous fermentation on BA accumulation is depicted in Figure 3. Tryptamine, histamine, phenylethylamine, tyramine, cadaverine, putrescine, spermine, and spermidine are a few of the common BAs found in food. The most common ones in meat are histamine, putrescine, tyramine, and cadaverine. These four BAs were detected throughout the fermentation process in both control and starter culture groups. Cadaverine content was highest, followed by tyramine, putrescine, and histamine in the control group. As fermentation time increased, the content of all BAs rose, except for histamine. The highest histamine levels in the control group reached 24.8 mg/kg on day 40, while the *LLS* starter culture group maintained levels below 5 mg/kg on day 60. In this study, biogenic amine levels—specifically histamine, putrescine, and cadaverine—in the *LLS* and S015 groups were substantially lower than those in the control group (*p* < 0.05). Tyramine concentrations showed no significant difference between the control and S015 groups (*p* > 0.05). In the later stages of fermentation, the histamine content of the control group showed a decreasing trend, while the pH increased significantly (Figure 4A). Histamine is the product of decarboxylation under the action of histidine decarboxylase, which is associated with pH, microorganisms, and free amino acids. The pH change may regulate the microbiota and affect histamine content (Komprda et al. 2007). These results indicate that inoculation with starter cultures significantly reduces the formation of most BAs compared to natural fermentation. The *LLS* starter culture exhibited stronger inhibition of BA accumulation than the S015.

During meat fermentation, proteases secreted by microorganisms break down proteins into amino acids, which are then decarboxylated to form BAs. Recent reports suggest that mixed starter cultures can effectively inhibit the growth of rapid amine‐producing bacteria in fermented products, thus reducing BA formation. Fifty strains of *S. xylosus* with amine oxidase activity were isolated from Italian sausage, six of which could reduce histamine content by 100% (Martuscelli et al. 2010). *L. casei* IFI‐CA 52 significantly reduced histamine and humutine in wine while inhibiting hydrogen sulfide production and reducing the pungent taste of sulfur dioxide (García‐Ruiz et al. 2011). In this study, the microbial dynamics within the *LLS* starter culture enhanced the relative abundance of LAB and diminished the presence of spoilage organisms, thereby potentially mitigating BA accumulation during fermentation.

### 3.3 Effect of the Starter Culture on pH, *a*_*w*_, and TVB‐N

The pH, a_w_, and TVB‐N values for the starter cultures are depicted in Figure 4. Initially, the pH was 5.90 across the control, *LLS*, and S015 samples. Subsequently, the pH levels in all groups exhibited a rapid decline, reaching their nadir on Day 40. By the end of the fermentation period, the pH levels registered at 5.20 for the control, 4.80 for *LLS*, and 5.15 for S015. A decreasing trend in a_w_ was observed in each group, with *LLS* recording the lowest value of 0.86 at the end of fermentation.

During meat fermentation, the acid production by microorganisms reduces the pH and water‐holding capacity of proteins. The acidifying environment (pH < 5.0) prevents the proliferation of putrefying microorganisms and prolongs the shelf life of meat products. Optimal pH value and low a_w_ improve flavor and color and extend product shelf life. *Lactobacilli* became the predominating strain, producing higher acid capability for lower pH and a_w_ values after inoculation with LAB, which could improve the quality of fermented meat.

Protein degradation and freshness in meat were assessed using TVB‐N values. TVB‐N values in all ham groups increased as fermentation progressed, with the control group increasing the fastest, followed by the S015 and *LLS* groups. These findings are in line with previous studies indicating that LAB starter cultures help reduce TVB‐N (Xiao et al. 2018).

### 3.4 Effect of the Starter Culture on Color and Texture

**FIGURE 5:**
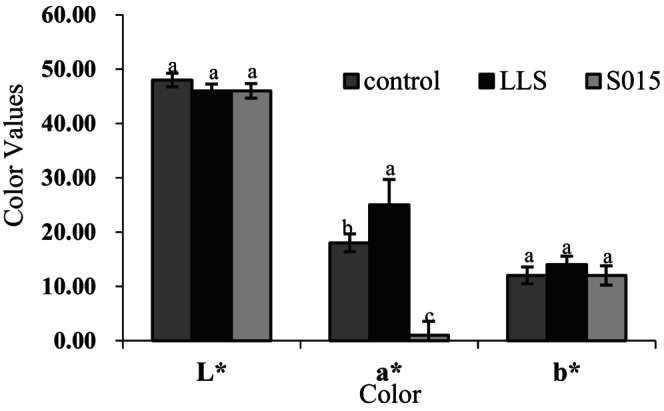
Effect of the starter culture on color. Different letters indicate significant differences in different groups at the same stage (*p* < 0.05).

Colorimetry of the hams was determined by three indicators: L*, a*, and b*. The L* value, indicating brightness, is associated with the moisture content of the sausages; the a* value, denoting redness, reflects the reddish hue of the samples; and the b* value, representing yellowness, increases with fat oxidation and protein denaturation. Differences in colorimetric values among the three groups are illustrated in Figure 5. The L* values in the starter culture groups were lower than those in the control, suggesting that the complex starter culture accelerated the fermentation process, leading to quicker water loss and reduced brightness of the ham (Chen et al. 2022). The a* value of the *LLS* group was significantly higher (*p* < 0.05) than that of the control and S015 group, indicating that LAB facilitated the conversion of nitrate to nitrite, which combined with myoglobin to form nitric oxide myoglobin, enhancing the red color of the ham. No significant difference was found in the b* values between the groups (*p* > 0.05).

Texture properties of the ham products at the end of fermentation are presented in Table 1. Relative to the control, both the *LLS* and S015 groups displayed significantly reduced hardness and chewiness. Conversely, the springiness in the *LLS* group surpassed that observed in both the control and S015 groups. These results align with those reported by Ren et al. 2002. LAB‐produced proteases denatured the proteins in the meat, increasing firmness. As fermentation progressed, the muscle in the ham degraded, loosening the tissues and enhancing muscle elasticity (Wang, Aziz, et al. 2022; Wang, Hou, et al. 2022).

**TABLE 1:** Texture property analysis of Jinhua ham.

	Hardness (N/mm)	Chewiness (N/mm·s)	Springiness (N/mm)
Control	12.23 ± 1.23^a^	50.54 ± 1.89^a^	5.67 ± 0.14^a^
LLS	9.65 ± 0.34^b^	45.98 ± 2.09^b^	7.86 ± 0.21^b^
S015	9.87 ± 0.26^b^	45.02 ± 1.34^b^	6.34 ± 0.37^c^

*Note:* Data show mean ± SD (*n* = 10); mean values with different letters are significantly different (*p* < 0.05).

### 3.5 Effect of Starter Culture on Volatile Substances

The Relative Odor Activity Value (ROAV) method was employed to identify key substances that modify the overall flavor, with results presented in Table 2. Differences were noted in the types of main flavor compounds among the groups, with the *LLS* group exhibiting the highest diversity: 5 acids, 14 aldehydes, 9 alcohols, 7 ketones, 5 esters, 3 pyrazines, 5 alkenes, and 5 others. This was followed by the S015 and control groups. Compared to the control, the starter cultures exhibited an increase in 11 volatile compounds. Degradation of proteins, fats, and carbohydrates through the action of microorganisms plays an important role in the production of free fatty acids and amino acids participating in the volatile compounds responsible for flavor. This enrichment of the flavor profile of Jinhua ham took place from its high microbial diversity present in *LLS* starter culture.

**TABLE 2:** Effect of starter culture on volatile substances of Jinhua ham.

No.	Volatile compound	ROAV		
		Control (μg/kg)	LLS (μg/kg)	S015 (μg/kg)
**Acids**				
1	Acetic acid	1.21 ± 0.66^a^	2.87 ± 0.12^b^	4.48 ± 0.52^c^
2	Dodecenoic acid	2.08 ± 0.02^a^	6.88 ± 0.07^b^	8.25 ± 0.02^c^
3	Undecylenic acid	1.67 ± 0.56^a^	1.76 ± 0.32^a^	1.63 ± 0.39^a^
4	Pelargonic acid	0.42 ± 0.01^a^	0.48 ± 0.56^a^	0.67 ± 0.56^b^
5	2‐Methylcaproic acid	0.15 ± 0.12^a^	0.14 ± 0.43^a^	0.10 ± 0.09^a^
**Alcohols**				
6	Ethanol	0.02 ± 0.17^a^	0.10 ± 0.01^b^	0.21 ± 0.02^c^
7	3‐Methylbutanol	0.45 ± 0.02^a^	2.19 ± 0.09^c^	1.87 ± 0.02^b^
8	3‐Methyl‐1‐butanol	0.05 ± 0.02^a^	4.19 ± 0.13^c^	2.76 ± 0.21^b^
9	1‐Hexanol	0.05 ± 0.02^a^	0.03 ± 0.11^a^	0.05 ± 0.23^a^
10	1‐Heptanol	1.19 ± 0.12^a^	1.12 ± 0.23^a^	0.34 ± 0.09^b^
11	1‐Octene‐3‐alcohol	12.17 ± 0.62^a^	36.17 ± 1.22^b^	15.23 ± 1.23^c^
12	2‐Ethyl‐1‐hexanol	0.96 ± 0.11^a^	3.92 ± 0.17^b^	0.67 ± 0.03^a^
13	1‐Octanol	0.07 ± 0.05^a^	0.07 ± 0.65^a^	0.08 ± 0.17^a^
14	2‐Hexyl‐1‐octanol	0.24 ± 0.11^a^	0.26 ± 0.09^a^	0.25 ± 0.17^a^
**Aldehydes**				
15	Enanthal	0.76 ± 0.15^c^	0.65 ± 0.35^b^	0.46 ± 0.06^a^
16	Nonyl aldehyde	0.10 ± 0.15^a^	0.12 ± 0.13^a^	0.09 ± 0.23^a^
17	Caprylaldehyde	0.06 ± 0.01^b^	0.06 ± 0.00^b^	0.04 ± 0.07^a^
18	Dapric aldehyde	0.07 ± 0.11^a^	0.34 ± 0.11^b^	0.28 ± 0.06^c^
19	Caproaldehyde	2.87 ± 0.12^b^	1.06 ± 0.09^a^	2.81 ± 0.34^b^
20	2‐Methyl‐propanal	0.34 ± 0.14^a^	1.67 ± 0.08^c^	1.23 ± 0.09^b^
21	2‐Methyl‐butyraldehyde	9.25 ± 0.56^a^	7.76 ± 0.47^b^	7.25 ± 1.28^b^
22	3‐Methyl‐butyraldehyde	1.09 ± 0.12^a^	1.11 ± 0.13^a^	1.09 ± 0.45^a^
23	Methyl thiopropionaldehyde	0.13 ± 0.09^a^	1.67 ± 0.08^b^	1.62 ± 0.35^b^
24	2‐Octene‐2‐aldehyde	0.20 ± 0.14^a^	0.15 ± 0.09^a^	0.22 ± 0.06^a^
25	Benzaldehyde	0.34 ± 1.04^a^	0.67 ± 2.02^b^	0.89 ± 0.08^a^
26	Nonanal	0.26 ± 0.11^a^	0.09 ± 0.07^a^	0.12 ± 0.09^a^
27	Lauraldehyde	1.22 ± 0.02^a^	4.98 ± 0.11^b^	4.25 ± 1.02^b^
28	13‐Methyltetradecral	1.65 ± 0.15^b^	1.34 ± 0.17^a^	1.26 ± 0.11^a^
**Ketones**				
29	2, 3‐Butanedione	0.22 ± 0.02^c^	3.78 ± 0.16^a^	1.09 ± 0.02^b^
30	3‐Hydroxydibutanone	ND^a^	ND^a^	5.76 ± 0.12^b^
31	1‐Hydroxy‐2‐diacetone	0.20 ± 0.08^a^	0.16 ± 0.01^a^	0.12 ± 0.12^a^
32	3‐Heptanone	1.25 ± 0.23^a^	1.15 ± 0.13^a^	1.26 ± 0.26^a^
33	2, 3‐Octanedione	1.15 ± 0.11^a^	1.08 ± 0.09^a^	1.12 ± 0.07^a^
34	L‐carvone	2.36 ± 0.21^b^	ND^a^	ND^a^
35	Methylketene	1.09 ± 0.08^b^	1.06 ± 0.07^a^	1.10 ± 0.10^a^
36	2‐Undecanone	2.36 ± 0.12^c^	2.19 ± 0.11^b^	1.65 ± 0.07^a^
37	5, 9‐Undecadienal‐2‐ketone	3.25 ± 0.15^a^	3.18 ± 0.18^a^	3.26 ± 0.45^a^
**Esters**				
38	Ethyl butyrate	1.87 ± 0.09^b^	1.25 ± 0.76^a^	3.34 ± 0.34^c^
39	Ethyl‐caproate	0.27 ± 0.11^a^	0.25 ± 0.18^a^	0.23 ± 0.65^a^
40	Ethyl‐caprate	1.87 ± 0.12^b^	1.76 ± 0.12^a^	1.98 ± 0.28^b^
41	Ethyl pelanoate	1.65 ± 1.23^a^	1.89 ± 0.98^b^	1.76 ± 0.12^b^
42	Ethyl hexadecate	2.78 ± 0.12^b^	2.98 ± 0.04^a^	2.87 ± 0.11^a^
**Pyrazines**				
43	Methylpyrazine	3.87 ± 0.67^c^	2.45 ± 0.67^b^	ND^a^
44	2, 6‐Dimethylpyrazine	ND^a^	1.32 ± 0.57^b^	0.03 ± 0.65^a^
45	2,3, 5‐Trimethylpyrazine	ND^a^	1.90 ± 0.45^c^	0.92 ± 0.14^b^
**Alkenes**				
46	Laurene	15.25 ± 2.67^b^	13.16 ± 4.12^b^	9.68 ± 2.67^a^
47	Styrene	ND^a^	4.89 ± 0.34^b^	ND^a^
48	2, 4‐Dimethyl‐1‐heptene	2.89 ± 0.23^b^	2.25 ± 0.22^b^	1.89 ± 1.01^a^
49	Undecane	ND^a^	1.02 ± 0.11^c^	ND^a^
50	2, 3‐Di‐tert‐butyl benzene	ND^a^	1.12 ± 0.34^b^	ND^a^
**Others**				
51	2,4‐Di‐tert‐butylphenol	ND^a^	1.68 ± 0.12^b^	ND^a^
52	2‐Amyl alkyl furan	ND^a^	2.87 ± 0.26^b^	ND^a^
53	m‐Cresol	ND^a^	2.56 ± 0.89^b^	ND^a^
54	Methoxyphenyloxime	ND^a^	0.21 ± 0.09^a^	ND^a^
55	Divinyl sulfide	ND^a^	ND^a^	1.74 ± 0.26^b^
56	3, 4‐Dihydroxy‐5‐methyldihydrofuran	1.76 ± 0.11^b^	0.25 ± 0.18^a^	1.75 ± 0.45^a^

*Note:* Data show the mean ± SD; mean values with different letters are significantly different (*P* < 0.05).

Abbreviation: ND, not detected.

Aldehydes are indicative of lipid oxidation and play an important role in flavor enhancement because of their low olfactory thresholds. These compounds mainly form through the oxidation of unsaturated fatty acids like oleic and linoleic acids, which are very important for flavor formation in dry‐cured meats such as Jinhua ham (Wang et al. 2020). Hexanal, benzaldehyde, and nonanal were the predominant aldehydes detected, with hexanal, a notable lipid oxidation product imparting a fresh, grassy flavor and serving as an indicator of lipid oxidation, presenting in the highest concentrations. The content of hexanal in the *LLS* group was the lowest and significantly reduced compared to the control and S015 groups (*p* < 0.05), indicating that the starter culture inhibited lipid oxidation. Alcohols, primarily produced as lipid oxidation byproducts, included 2‐ethyl‐1‐hexanol with a floral aroma, 1‐octen‐3‐alcohol with a mushroom aroma, and 3‐methyl‐1‐butanol with a fruity aroma (Wang, Aziz, et al. 2022; Wang, Hou, et al. 2022); their concentrations in the *LLS* group were significantly higher than in the control and S015 groups (*p* < 0.05), especially for 1‐octen‐3‐alcohol at 36.17 ± 1.22 μg/kg. 2,6‐dimethylpyrazine and 2,3,5‐trimethylpyrazine, reminiscent of baked potatoes, are important flavor compounds in ham (Liu et al. 2020). While undetectable in the control group, these compounds were more prevalent in the *LLS* group than in the S015 group. This result indicates that the LAB starter culture is more conducive to the formation of volatile compounds in hams than natural fermentation, with *LLS* proving superior to the commercial fermenter S015 for enhancing flavor compounds, suggesting a potential advantage of *LLS* as a starter culture for Jinhua ham.

### 3.6 Sensory Evaluation

**FIGURE 6:**
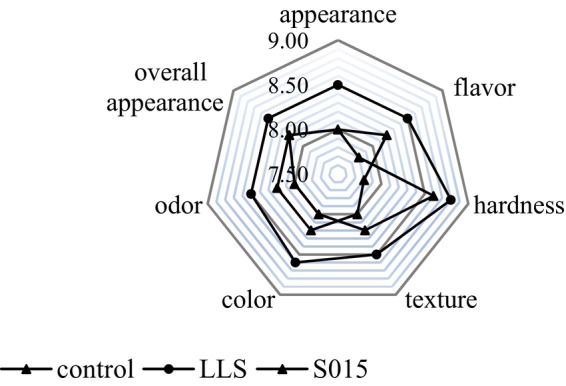
Comparative sensory characteristics of Jinhua ham with different starter cultures.

A radar map was used to evaluate the sensory properties of Jinhua ham subjected to spontaneous fermentation and those inoculated with starter cultures, as depicted in Figure 6. At the end of fermentation, sensory analysis of various Jinhua ham samples was conducted, measuring average scores for color, hardness, overall acceptability, flavor, odor, and texture. The *LLS* group scored higher than the control by +0.7 points for flavor, +0.5 points for appearance, and + 0.6 points for texture. Overall, average scores for the *LLS* group were higher than those for the S015 group, indicating superior sensory qualities in hams inoculated with the *LLS* starter culture.

## 4 Conclusions

In the *LLS* starter culture samples, higher relative abundances of the probiotics *Staphylococcus* and *Lactobacillaceae* and lower levels of the spoilage bacteria *Psychrobacteraceae* and *Carnobaciliaceae* were observed. The concentrations of histamine, putrescine, tyramine, and cadaverine in the *LLS* group showed stronger inhibition than those in the control and the commercial starter S015. Hams inoculated with the *LLS* starter culture also exhibited lower pH, a_w_, and TVBN. Additionally, the *LLS* samples contained 11 additional volatile compounds and a greater variety of flavors compared to the control, resulting in favorable sensory evaluations and physicochemical properties. These findings suggest that a mixed starter culture consisting of *Lactiplantibacillus plantarum, Lactilactobacillus sakei*, and *S. xylosus* has the potential to reduce BAs and enhance sensory quality in Jinhua ham.

## Author Contributions

**Lin Chen**: investigation (equal), methodology (equal), writing – original draft (equal). **Lu Huang**: investigation (equal), methodology (equal). **WuMei Yu:** methodology (equal), writing – original draft (equal). **QingYu Zhang:** formal analysis (equal), validation (equal).

## Ethics Statement

The sensory evaluation was reviewed and approved by The Ethics Committee of Zhejiang Chinese Medical University, China (JN. No 202410668), and this experiment was conducted according to the European Community guidelines.

## Consent

All persons gave their informed consent prior to their inclusion in the study.

## Conflicts of Interest

The authors declare no conflicts of interest.

## Data Availability

The data that support the findings of this study are available from the corresponding author upon reasonable request.
